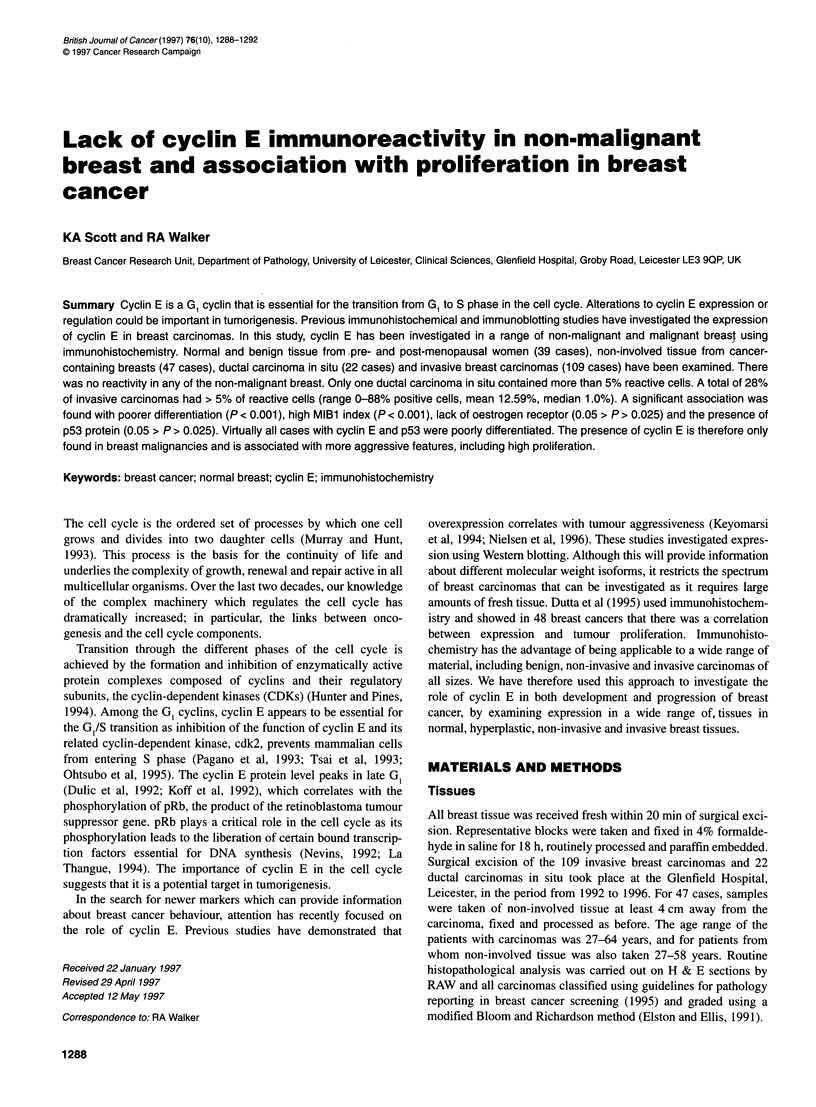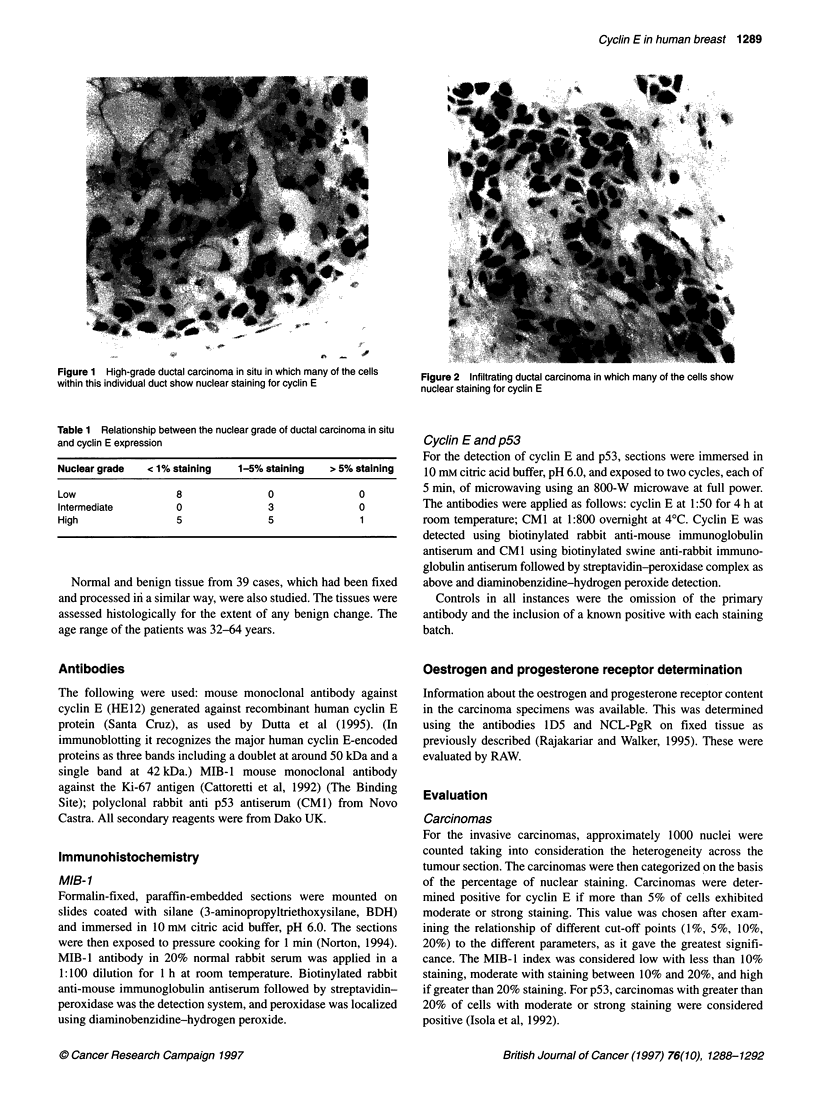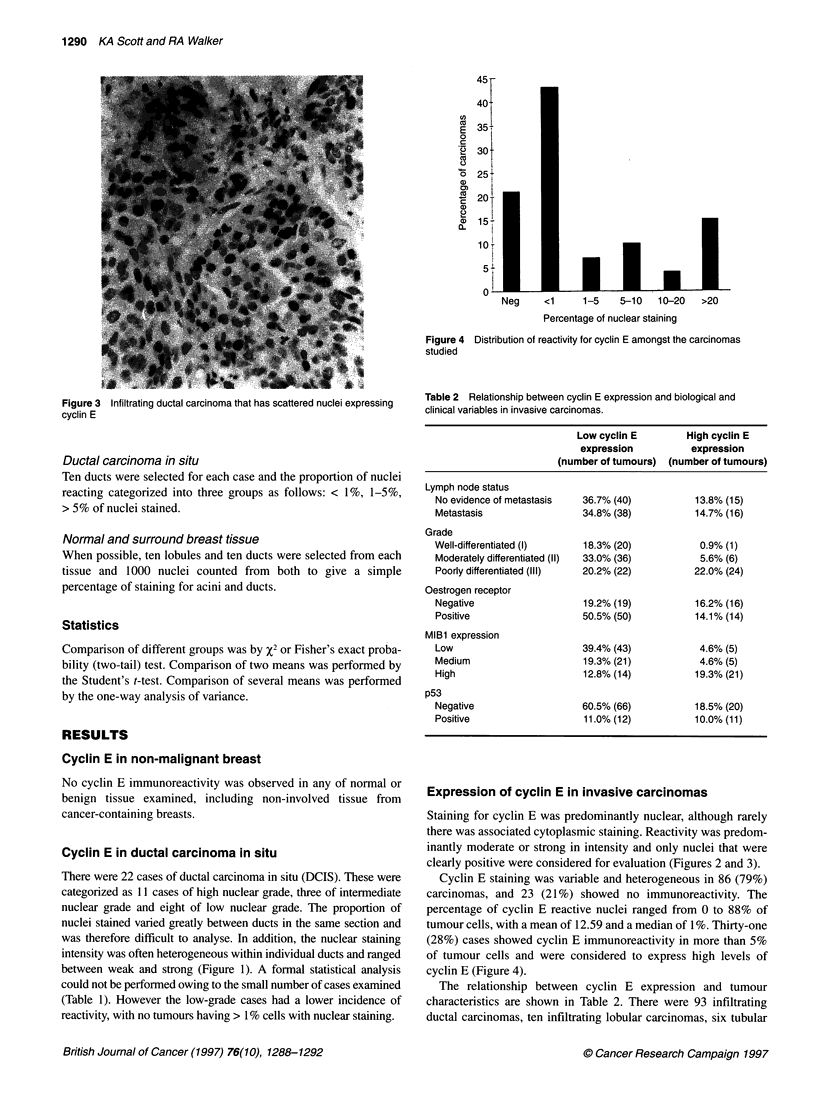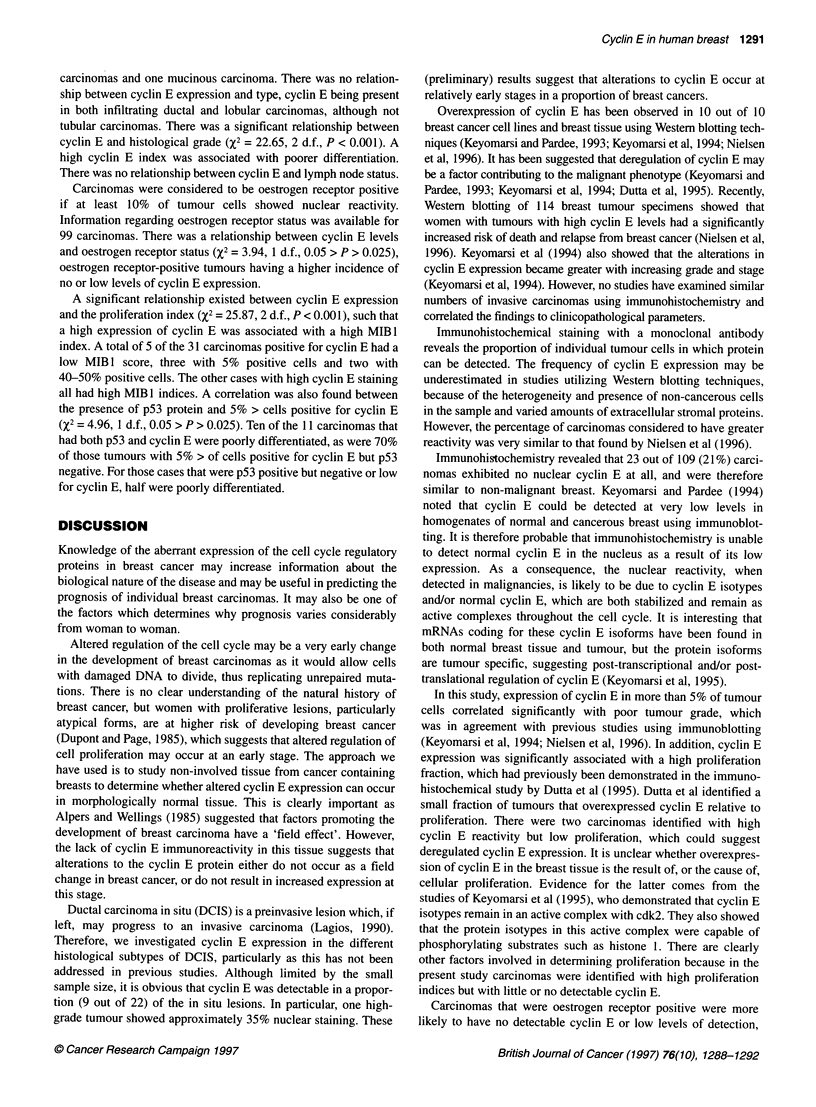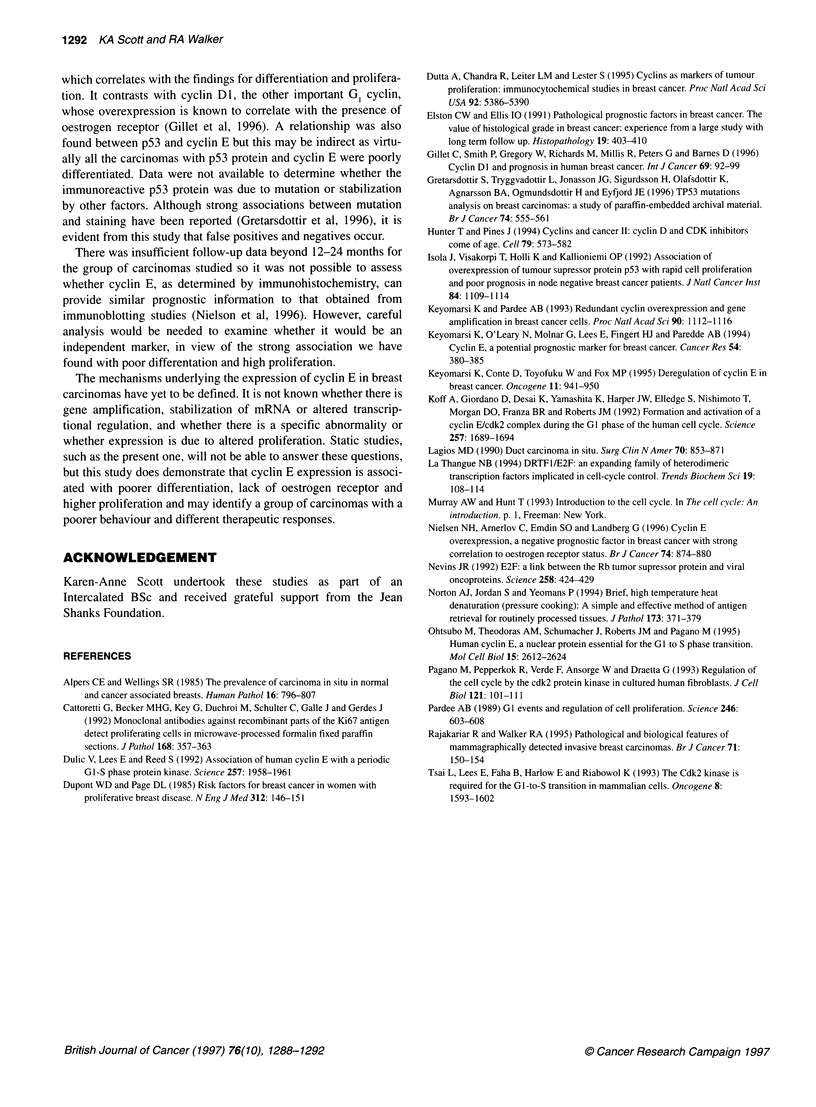# Lack of cyclin E immunoreactivity in non-malignant breast and association with proliferation in breast cancer.

**DOI:** 10.1038/bjc.1997.550

**Published:** 1997

**Authors:** K. A. Scott, R. A. Walker

**Affiliations:** Department of Pathology, University of Leicester, Clinical Sciences, Glenfield Hospital, UK.

## Abstract

**Images:**


British Journal of Cancer (1997) **76**(10), 1288–1292
© 1997 Cancer Research Campaign

### Lack of cyclin E immunoreactivity in non-malignant breast and association with proliferation in breast cancer

**KA Scott and RA Walker**

Breast Cancer Research Unit, Department of Pathology, University of Leicester, Clinical Sciences, Glenfield Hospital, Groby Road, Leicester LE3 9QP, UK

**Summary** Cyclin E is a $G_1$ cyclin that is essential for the transition from $G_1$ to S phase in the cell cycle. Alterations to cyclin E expression or regulation could be important in tumorigenesis. Previous immunohistochemical and immunoblotting studies have investigated the expression of cyclin E in breast carcinomas. In this study, cyclin E has been investigated in a range of non-malignant and malignant breast using immunohistochemistry. Normal and benign tissue from pre- and post-menopausal women (39 cases), non-involved tissue from cancer-containing breasts (47 cases), ductal carcinoma in situ (22 cases) and invasive breast carcinomas (109 cases) have been examined. There was no reactivity in any of the non-malignant breast. Only one ductal carcinoma in situ contained more than 5% reactive cells. A total of 28% of invasive carcinomas had > 5% of reactive cells (range 0–88% positive cells, mean 12.59%, median 1.0%). A significant association was found with poorer differentiation ($P < 0.001$), high MIB1 index ($P < 0.001$), lack of oestrogen receptor ($0.05 > P > 0.025$) and the presence of p53 protein ($0.05 > P > 0.025$). Virtually all cases with cyclin E and p53 were poorly differentiated. The presence of cyclin E is therefore only found in breast malignancies and is associated with more aggressive features, including high proliferation.

**Keywords:** breast cancer; normal breast; cyclin E; immunohistochemistry

The cell cycle is the ordered set of processes by which one cell grows and divides into two daughter cells (Murray and Hunt, 1993). This process is the basis for the continuity of life and underlies the complexity of growth, renewal and repair active in all multicellular organisms. Over the last two decades, our knowledge of the complex machinery which regulates the cell cycle has dramatically increased; in particular, the links between oncogenesis and the cell cycle components.

Transition through the different phases of the cell cycle is achieved by the formation and inhibition of enzymatically active protein complexes composed of cyclins and their regulatory subunits, the cyclin-dependent kinases (CDKs) (Hunter and Pines, 1994). Among the $G_1$ cyclins, cyclin E appears to be essential for the $G_1$/S transition as inhibition of the function of cyclin E and its related cyclin-dependent kinase, cdk2, prevents mammalian cells from entering S phase (Pagano et al, 1993; Tsai et al, 1993; Ohtsubo et al, 1995). The cyclin E protein level peaks in late $G_1$ (Dulic et al, 1992; Koff et al, 1992), which correlates with the phosphorylation of pRb, the product of the retinoblastoma tumour suppressor gene. pRb plays a critical role in the cell cycle as its phosphorylation leads to the liberation of certain bound transcription factors essential for DNA synthesis (Nevins, 1992; La Thangue, 1994). The importance of cyclin E in the cell cycle suggests that it is a potential target in tumorigenesis.

In the search for newer markers which can provide information about breast cancer behaviour, attention has recently focused on the role of cyclin E. Previous studies have demonstrated that overexpression correlates with tumour aggressiveness (Keyomarsi et al, 1994; Nielsen et al, 1996). These studies investigated expression using Western blotting. Although this will provide information about different molecular weight isoforms, it restricts the spectrum of breast carcinomas that can be investigated as it requires large amounts of fresh tissue. Dutta et al (1995) used immunohistochemistry and showed in 48 breast cancers that there was a correlation between expression and tumour proliferation. Immunohistochemistry has the advantage of being applicable to a wide range of material, including benign, non-invasive and invasive carcinomas of all sizes. We have therefore used this approach to investigate the role of cyclin E in both development and progression of breast cancer, by examining expression in a wide range of tissues in normal, hyperplastic, non-invasive and invasive breast tissues.

*Received 22 January 1997*
*Revised 29 April 1997*
*Accepted 12 May 1997*

*Correspondence to:* RA Walker

### MATERIALS AND METHODS

### Tissues

All breast tissue was received fresh within 20 min of surgical excision. Representative blocks were taken and fixed in 4% formaldehyde in saline for 18 h, routinely processed and paraffin embedded. Surgical excision of the 109 invasive breast carcinomas and 22 ductal carcinomas in situ took place at the Glenfield Hospital, Leicester, in the period from 1992 to 1996. For 47 cases, samples were taken of non-involved tissue at least 4 cm away from the carcinoma, fixed and processed as before. The age range of the patients with carcinomas was 27–64 years, and for patients from whom non-involved tissue was also taken 27–58 years. Routine histopathological analysis was carried out on H & E sections by RAW and all carcinomas classified using guidelines for pathology reporting in breast cancer screening (1995) and graded using a modified Bloom and Richardson method (Elston and Ellis, 1991).

**Figure 1** High-grade ductal carcinoma in situ in which many of the cells within this individual duct show nuclear staining for cyclin E

**Figure 2** Infiltrating ductal carcinoma in which many of the cells show nuclear staining for cyclin E

**Table 1** Relationship between the nuclear grade of ductal carcinoma in situ and cyclin E expression

| Nuclear grade | < 1% staining | 1–5% staining | > 5% staining |
|---|---|---|---|
| Low | 8 | 0 | 0 |
| Intermediate | 0 | 3 | 0 |
| High | 5 | 5 | 1 |

Normal and benign tissue from 39 cases, which had been fixed and processed in a similar way, were also studied. The tissues were assessed histologically for the extent of any benign change. The age range of the patients was 32–64 years.

### Antibodies

The following were used: mouse monoclonal antibody against cyclin E (HE12) generated against recombinant human cyclin E protein (Santa Cruz), as used by Dutta et al (1995). (In immunoblotting it recognizes the major human cyclin E-encoded proteins as three bands including a doublet at around 50 kDa and a single band at 42 kDa.) MIB-1 mouse monoclonal antibody against the Ki-67 antigen (Cattoretti et al, 1992) (The Binding Site); polyclonal rabbit anti p53 antiserum (CM1) from Novo Castra. All secondary reagents were from Dako UK.

### Immunohistochemistry

### *MIB-1*

Formalin-fixed, paraffin-embedded sections were mounted on slides coated with silane (3-aminopropyltriethoxysilane, BDH) and immersed in 10 mM citric acid buffer, pH 6.0. The sections were then exposed to pressure cooking for 1 min (Norton, 1994). MIB-1 antibody in 20% normal rabbit serum was applied in a 1:100 dilution for 1 h at room temperature. Biotinylated rabbit anti-mouse immunoglobulin antiserum followed by streptavidin–peroxidase was the detection system, and peroxidase was localized using diaminobenzidine–hydrogen peroxide.

### *Cyclin E and p53*

For the detection of cyclin E and p53, sections were immersed in 10 mM citric acid buffer, pH 6.0, and exposed to two cycles, each of 5 min, of microwaving using an 800-W microwave at full power. The antibodies were applied as follows: cyclin E at 1:50 for 4 h at room temperature; CM1 at 1:800 overnight at 4°C. Cyclin E was detected using biotinylated rabbit anti-mouse immunoglobulin antiserum and CM1 using biotinylated swine anti-rabbit immunoglobulin antiserum followed by streptavidin–peroxidase complex as above and diaminobenzidine–hydrogen peroxide detection.

Controls in all instances were the omission of the primary antibody and the inclusion of a known positive with each staining batch.

### Oestrogen and progesterone receptor determination

Information about the oestrogen and progesterone receptor content in the carcinoma specimens was available. This was determined using the antibodies 1D5 and NCL-PgR on fixed tissue as previously described (Rajakariar and Walker, 1995). These were evaluated by RAW.

### Evaluation

### *Carcinomas*

For the invasive carcinomas, approximately 1000 nuclei were counted taking into consideration the heterogeneity across the tumour section. The carcinomas were then categorized on the basis of the percentage of nuclear staining. Carcinomas were determined positive for cyclin E if more than 5% of cells exhibited moderate or strong staining. This value was chosen after examining the relationship of different cut-off points (1%, 5%, 10%, 20%) to the different parameters, as it gave the greatest significance. The MIB-1 index was considered low with less than 10% staining, moderate with staining between 10% and 20%, and high if greater than 20% staining. For p53, carcinomas with greater than 20% of cells with moderate or strong staining were considered positive (Isola et al, 1992).

© Cancer Research Campaign 1997

Figure 3 Infiltrating ductal carcinoma that has scattered nuclei expressing cyclin E

Figure 4 Distribution of reactivity for cyclin E amongst the carcinomas studied

Table 2 Relationship between cyclin E expression and biological and clinical variables in invasive carcinomas.

| | Low cyclin E expression (number of tumours) | High cyclin E expression (number of tumours) |
|---|---|---|
| Lymph node status | | |
| No evidence of metastasis | 36.7% (40) | 13.8% (15) |
| Metastasis | 34.8% (38) | 14.7% (16) |
| Grade | | |
| Well-differentiated (I) | 18.3% (20) | 0.9% (1) |
| Moderately differentiated (II) | 33.0% (36) | 5.6% (6) |
| Poorly differentiated (III) | 20.2% (22) | 22.0% (24) |
| Oestrogen receptor | | |
| Negative | 19.2% (19) | 16.2% (16) |
| Positive | 50.5% (50) | 14.1% (14) |
| MIB1 expression | | |
| Low | 39.4% (43) | 4.6% (5) |
| Medium | 19.3% (21) | 4.6% (5) |
| High | 12.8% (14) | 19.3% (21) |
| p53 | | |
| Negative | 60.5% (66) | 18.5% (20) |
| Positive | 11.0% (12) | 10.0% (11) |

### *Ductal carcinoma in situ*

Ten ducts were selected for each case and the proportion of nuclei reacting categorized into three groups as follows: < 1%, 1–5%, > 5% of nuclei stained.

### *Normal and surround breast tissue*

When possible, ten lobules and ten ducts were selected from each tissue and 1000 nuclei counted from both to give a simple percentage of staining for acini and ducts.

### Statistics

Comparison of different groups was by $\chi^2$ or Fisher's exact probability (two-tail) test. Comparison of two means was performed by the Student's *t*-test. Comparison of several means was performed by the one-way analysis of variance.

### RESULTS

### Cyclin E in non-malignant breast

No cyclin E immunoreactivity was observed in any of normal or benign tissue examined, including non-involved tissue from cancer-containing breasts.

### Cyclin E in ductal carcinoma in situ

There were 22 cases of ductal carcinoma in situ (DCIS). These were categorized as 11 cases of high nuclear grade, three of intermediate nuclear grade and eight of low nuclear grade. The proportion of nuclei stained varied greatly between ducts in the same section and was therefore difficult to analyse. In addition, the nuclear staining intensity was often heterogeneous within individual ducts and ranged between weak and strong (Figure 1). A formal statistical analysis could not be performed owing to the small number of cases examined (Table 1). However the low-grade cases had a lower incidence of reactivity, with no tumours having > 1% cells with nuclear staining.

### Expression of cyclin E in invasive carcinomas

Staining for cyclin E was predominantly nuclear, although rarely there was associated cytoplasmic staining. Reactivity was predominantly moderate or strong in intensity and only nuclei that were clearly positive were considered for evaluation (Figures 2 and 3).

Cyclin E staining was variable and heterogeneous in 86 (79%) carcinomas, and 23 (21%) showed no immunoreactivity. The percentage of cyclin E reactive nuclei ranged from 0 to 88% of tumour cells, with a mean of 12.59 and a median of 1%. Thirty-one (28%) cases showed cyclin E immunoreactivity in more than 5% of tumour cells and were considered to express high levels of cyclin E (Figure 4).

The relationship between cyclin E expression and tumour characteristics are shown in Table 2. There were 93 infiltrating ductal carcinomas, ten infiltrating lobular carcinomas, six tubular

© Cancer Research Campaign 1997

carcinomas and one mucinous carcinoma. There was no relationship between cyclin E expression and type, cyclin E being present in both infiltrating ductal and lobular carcinomas, although not tubular carcinomas. There was a significant relationship between cyclin E and histological grade ($\chi^2 = 22.65$, 2 d.f., $P < 0.001$). A high cyclin E index was associated with poorer differentiation. There was no relationship between cyclin E and lymph node status.

Carcinomas were considered to be oestrogen receptor positive if at least 10% of tumour cells showed nuclear reactivity. Information regarding oestrogen receptor status was available for 99 carcinomas. There was a relationship between cyclin E levels and oestrogen receptor status ($\chi^2 = 3.94$, 1 d.f., $0.05 > P > 0.025$), oestrogen receptor-positive tumours having a higher incidence of no or low levels of cyclin E expression.

A significant relationship existed between cyclin E expression and the proliferation index ($\chi^2 = 25.87$, 2 d.f., $P < 0.001$), such that a high expression of cyclin E was associated with a high MIB1 index. A total of 5 of the 31 carcinomas positive for cyclin E had a low MIB1 score, three with 5% positive cells and two with 40–50% positive cells. The other cases with high cyclin E staining all had high MIB1 indices. A correlation was also found between the presence of p53 protein and 5% > cells positive for cyclin E ($\chi^2 = 4.96$, 1 d.f., $0.05 > P > 0.025$). Ten of the 11 carcinomas that had both p53 and cyclin E were poorly differentiated, as were 70% of those tumours with 5% > of cells positive for cyclin E but p53 negative. For those cases that were p53 positive but negative or low for cyclin E, half were poorly differentiated.

### DISCUSSION

Knowledge of the aberrant expression of the cell cycle regulatory proteins in breast cancer may increase information about the biological nature of the disease and may be useful in predicting the prognosis of individual breast carcinomas. It may also be one of the factors which determines why prognosis varies considerably from woman to woman.

Altered regulation of the cell cycle may be a very early change in the development of breast carcinomas as it would allow cells with damaged DNA to divide, thus replicating unrepaired mutations. There is no clear understanding of the natural history of breast cancer, but women with proliferative lesions, particularly atypical forms, are at higher risk of developing breast cancer (Dupont and Page, 1985), which suggests that altered regulation of cell proliferation may occur at an early stage. The approach we have used is to study non-involved tissue from cancer containing breasts to determine whether altered cyclin E expression can occur in morphologically normal tissue. This is clearly important as Alpers and Wellings (1985) suggested that factors promoting the development of breast carcinoma have a 'field effect'. However, the lack of cyclin E immunoreactivity in this tissue suggests that alterations to the cyclin E protein either do not occur as a field change in breast cancer, or do not result in increased expression at this stage.

Ductal carcinoma in situ (DCIS) is a preinvasive lesion which, if left, may progress to an invasive carcinoma (Lagios, 1990). Therefore, we investigated cyclin E expression in the different histological subtypes of DCIS, particularly as this has not been addressed in previous studies. Although limited by the small sample size, it is obvious that cyclin E was detectable in a proportion (9 out of 22) of the in situ lesions. In particular, one high-grade tumour showed approximately 35% nuclear staining. These (preliminary) results suggest that alterations to cyclin E occur at relatively early stages in a proportion of breast cancers.

Overexpression of cyclin E has been observed in 10 out of 10 breast cancer cell lines and breast tissue using Western blotting techniques (Keyomarsi and Pardee, 1993; Keyomarsi et al, 1994; Nielsen et al, 1996). It has been suggested that deregulation of cyclin E may be a factor contributing to the malignant phenotype (Keyomarsi and Pardee, 1993; Keyomarsi et al, 1994; Dutta et al, 1995). Recently, Western blotting of 114 breast tumour specimens showed that women with tumours with high cyclin E levels had a significantly increased risk of death and relapse from breast cancer (Nielsen et al, 1996). Keyomarsi et al (1994) also showed that the alterations in cyclin E expression became greater with increasing grade and stage (Keyomarsi et al, 1994). However, no studies have examined similar numbers of invasive carcinomas using immunohistochemistry and correlated the findings to clinicopathological parameters.

Immunohistochemical staining with a monoclonal antibody reveals the proportion of individual tumour cells in which protein can be detected. The frequency of cyclin E expression may be underestimated in studies utilizing Western blotting techniques, because of the heterogeneity and presence of non-cancerous cells in the sample and varied amounts of extracellular stromal proteins. However, the percentage of carcinomas considered to have greater reactivity was very similar to that found by Nielsen et al (1996).

Immunohistochemistry revealed that 23 out of 109 (21%) carcinomas exhibited no nuclear cyclin E at all, and were therefore similar to non-malignant breast. Keyomarsi and Pardee (1994) noted that cyclin E could be detected at very low levels in homogenates of normal and cancerous breast using immunoblotting. It is therefore probable that immunohistochemistry is unable to detect normal cyclin E in the nucleus as a result of its low expression. As a consequence, the nuclear reactivity, when detected in malignancies, is likely to be due to cyclin E isotypes and/or normal cyclin E, which are both stabilized and remain as active complexes throughout the cell cycle. It is interesting that mRNAs coding for these cyclin E isoforms have been found in both normal breast tissue and tumour, but the protein isoforms are tumour specific, suggesting post-transcriptional and/or post-translational regulation of cyclin E (Keyomarsi et al, 1995).

In this study, expression of cyclin E in more than 5% of tumour cells correlated significantly with poor tumour grade, which was in agreement with previous studies using immunoblotting (Keyomarsi et al, 1994; Nielsen et al, 1996). In addition, cyclin E expression was significantly associated with a high proliferation fraction, which had previously been demonstrated in the immunohistochemical study by Dutta et al (1995). Dutta et al identified a small fraction of tumours that overexpressed cyclin E relative to proliferation. There were two carcinomas identified with high cyclin E reactivity but low proliferation, which could suggest deregulated cyclin E expression. It is unclear whether overexpression of cyclin E in the breast tissue is the result of, or the cause of, cellular proliferation. Evidence for the latter comes from the studies of Keyomarsi et al (1995), who demonstrated that cyclin E isotypes remain in an active complex with cdk2. They also showed that the protein isotypes in this active complex were capable of phosphorylating substrates such as histone 1. There are clearly other factors involved in determining proliferation because in the present study carcinomas were identified with high proliferation indices but with little or no detectable cyclin E.

Carcinomas that were oestrogen receptor positive were more likely to have no detectable cyclin E or low levels of detection,

© Cancer Research Campaign 1997

which correlates with the findings for differentiation and proliferation. It contrasts with cyclin D1, the other important $G_1$ cyclin, whose overexpression is known to correlate with the presence of oestrogen receptor (Gillet et al, 1996). A relationship was also found between p53 and cyclin E but this may be indirect as virtually all the carcinomas with p53 protein and cyclin E were poorly differentiated. Data were not available to determine whether the immunoreactive p53 protein was due to mutation or stabilization by other factors. Although strong associations between mutation and staining have been reported (Gretarsdottir et al, 1996), it is evident from this study that false positives and negatives occur.

There was insufficient follow-up data beyond 12–24 months for the group of carcinomas studied so it was not possible to assess whether cyclin E, as determined by immunohistochemistry, can provide similar prognostic information to that obtained from immunoblotting studies (Nielson et al, 1996). However, careful analysis would be needed to examine whether it would be an independent marker, in view of the strong association we have found with poor differentation and high proliferation.

The mechanisms underlying the expression of cyclin E in breast carcinomas have yet to be defined. It is not known whether there is gene amplification, stabilization of mRNA or altered transcriptional regulation, and whether there is a specific abnormality or whether expression is due to altered proliferation. Static studies, such as the present one, will not be able to answer these questions, but this study does demonstrate that cyclin E expression is associated with poorer differentiation, lack of oestrogen receptor and higher proliferation and may identify a group of carcinomas with a poorer behaviour and different therapeutic responses.

### ACKNOWLEDGEMENT

Karen-Anne Scott undertook these studies as part of an Intercalated BSc and received grateful support from the Jean Shanks Foundation.

### REFERENCES

Alpers CE and Wellings SR (1985) The prevalence of carcinoma in situ in normal and cancer associated breasts. *Human Pathol* **16**: 796–807

Cattoretti G, Becker MHG, Key G, Duchroi M, Schulter C, Galle J and Gerdes J (1992) Monoclonal antibodies against recombinant parts of the Ki67 antigen detect proliferating cells in microwave-processed formalin fixed paraffin sections. *J Pathol* **168**: 357–363

Dulic V, Lees E and Reed S (1992) Association of human cyclin E with a periodic G1-S phase protein kinase. *Science* **257**: 1958–1961

Dupont WD and Page DL (1985) Risk factors for breast cancer in women with proliferative breast disease. *N Eng J Med* **312**: 146–151

Dutta A, Chandra R, Leiter LM and Lester S (1995) Cyclins as markers of tumour proliferation: immunocytochemical studies in breast cancer. *Proc Natl Acad Sci USA* **92**: 5386–5390

Elston CW and Ellis IO (1991) Pathological prognostic factors in breast cancer. The value of histological grade in breast cancer: experience from a large study with long term follow up. *Histopathology* **19**: 403–410

Gillet C, Smith P, Gregory W, Richards M, Millis R, Peters G and Barnes D (1996) Cyclin D1 and prognosis in human breast cancer. *Int J Cancer* **69**: 92–99

Gretarsdottir S, Tryggvadottir L, Jonasson JG, Sigurdsson H, Olafsdottir K, Agnarsson BA, Ogmundsdottir H and Eyfjord JE (1996) TP53 mutations analysis on breast carcinomas: a study of paraffin-embedded archival material. *Br J Cancer* **74**: 555–561

Hunter T and Pines J (1994) Cyclins and cancer II: cyclin D and CDK inhibitors come of age. *Cell* **79**: 573–582

Isola J, Visakorpi T, Holli K and Kallioniemi OP (1992) Association of overexpression of tumour supressor protein p53 with rapid cell proliferation and poor prognosis in node negative breast cancer patients. *J Natl Cancer Inst* **84**: 1109–1114

Keyomarsi K and Pardee AB (1993) Redundant cyclin overexpression and gene amplification in breast cancer cells. *Proc Natl Acad Sci* **90**: 1112–1116

Keyomarsi K, O'Leary N, Molnar G, Lees E, Fingert HJ and Paredde AB (1994) Cyclin E, a potential prognostic marker for breast cancer. *Cancer Res* **54**: 380–385

Keyomarsi K, Conte D, Toyofuku W and Fox MP (1995) Deregulation of cyclin E in breast cancer. *Oncogene* **11**: 941–950

Koff A, Giordano D, Desai K, Yamashita K, Harper JW, Elledge S, Nishimoto T, Morgan DO, Franza BR and Roberts JM (1992) Formation and activation of a cyclin E/cdk2 complex during the G1 phase of the human cell cycle. *Science* **257**: 1689–1694

Lagios MD (1990) Duct carcinoma in situ. *Surg Clin N Amer* **70**: 853–871

La Thangue NB (1994) DRTF1/E2F: an expanding family of heterodimeric transcription factors implicated in cell-cycle control. *Trends Biochem Sci* **19**: 108–114

Murray AW and Hunt T (1993) Introduction to the cell cycle. In *The cell cycle: An introduction*. p. 1, Freeman: New York.

Nielsen NH, Arnerlov C, Emdin SO and Landberg G (1996) Cyclin E overexpression, a negative prognostic factor in breast cancer with strong correlation to oestrogen receptor status. *Br J Cancer* **74**: 874–880

Nevins JR (1992) E2F: a link between the Rb tumor supressor protein and viral oncoproteins. *Science* **258**: 424–429

Norton AJ, Jordan S and Yeomans P (1994) Brief, high temperature heat denaturation (pressure cooking): A simple and effective method of antigen retrieval for routinely processed tissues. *J Pathol* **173**: 371–379

Ohtsubo M, Theodoras AM, Schumacher J, Roberts JM and Pagano M (1995) Human cyclin E, a nuclear protein essential for the G1 to S phase transition. *Mol Cell Biol* **15**: 2612–2624

Pagano M, Pepperkok R, Verde F, Ansorge W and Draetta G (1993) Regulation of the cell cycle by the cdk2 protein kinase in cultured human fibroblasts. *J Cell Biol* **121**: 101–111

Pardee AB (1989) G1 events and regulation of cell proliferation. *Science* **246**: 603–608

Rajakariar R and Walker RA (1995) Pathological and biological features of mammagraphically detected invasive breast carcinomas. *Br J Cancer* **71**: 150–154

Tsai L, Lees E, Faha B, Harlow E and Riabowol K (1993) The Cdk2 kinase is required for the G1-to-S transition in mammalian cells. *Oncogene* **8**: 1593–1602

 © Cancer Research Campaign 1997